# Targeting and killing glioblastoma with monoclonal antibody to *O*-acetyl GD2 ganglioside

**DOI:** 10.18632/oncotarget.9226

**Published:** 2016-05-09

**Authors:** Julien Fleurence, Denis Cochonneau, Sophie Fougeray, Lisa Oliver, Fanny Geraldo, Mickaël Terme, Mylène Dorvillius, Delphine Loussouarn, François Vallette, François Paris, Stéphane Birklé

**Affiliations:** ^1^ INSERM U892, Centre de Recherche en Cancérologie de Nantes-Angers, Institut de Recherche en Santé de l'Université de Nantes, Nantes, France; ^2^ CNRS 6299, Centre de Recherche en Cancérologie de Nantes-Angers, Institut de Recherche en Santé de l'Université de Nantes, Nantes, France; ^3^ OGD2 Pharma, Institut de Recherche en Santé de l'Université de Nantes, Nantes, France; ^4^ Université de Nantes, UFR des Sciences Pharmaceutiques et Biologiques, Nantes, France; ^5^ Centre Hospitalier Universitaire de Nantes, Nantes, France; ^6^ LaBCT, Institut de Cancérologie de l'Ouest-René Gauducheau, Saint-Herblain, France

**Keywords:** glioblastoma, ganglioside, immunotherapy

## Abstract

There are still unmet medical needs in the treatment of glioblastoma, the most common and the most aggressive glioma of all brain tumors. Here, we found that *O*-acetyl GD2 is expressed in surgically resected human glioblastoma tissue. In addition, we demonstrated that 8B6 monoclonal antibody specific for *O*-acetylat GD2 could effectively inhibit glioblastoma cell proliferation *in vitro* and *in vivo*. Taken together, these results indicate that *O*-acetylated GD2 represents a novel antigen for immunotherapeutic-based treatment of high-grade gliomas.

## INTRODUCTION

Glioblastoma multiforme (GBM, WHO grade IV) is the most common primary brain tumor and it is invariably fatal [1]. Surgical resection combined with non-specific standard of care temozolomide chemotherapy and radiotherapy fail to eliminate completely tumor cells, resulting in a poor prognosis of less than 15 months in patients with this cancer [2]. Major reasons for treatment failure include the highly infiltrative growth pattern of GBM and chemo- and radio-resistance [3, 4]. Consequently, new effective strategies that can eliminate the residual tumor cells are urgently needed. Immunotherapy offers a precise approach for specifically targeting residual glioblastoma cells with reduced risk of collateral toxicity [5]. Some suitable glioma-specific cell surface antigens have been identified, such as the EGF receptor (EGFR) and its variant EGFRvIII, HER2/neu, IL-13 receptor alpha chain 2 [6], and the EPH receptor A2 (EphA2) [7]. Malignant gliomas are, however, highly heterogeneous tumors. This property gives them the capacity for immune escape, with the subsequent high-risk of recurrence of antigen-negative tumors [8]. Given these limitations, the identification of new tumor-specific antigens is necessary.

We have previously generated a mouse monoclonal antibody (mAb) that is specific for the *O*-acetylated derivative of the neuroblastoma-associated tumor antigen ganglioside GD2 [9]. Gangliosides represent a family of sialic acid-containing glycosphingolipids that are anchored in the plasma membrane by a lipophilic ceramide segment, with the antigenic carbohydrate section extending into the extracellular region. Through protein binding, gangliosides activate responsiveness of various signaling proteins (for a review see [10]). GD2, a b-series disialoganglioside, is highly expressed in neuroblastoma and several mAbs against GD2 have been developed in treatment of this cancer [11]. GD2 is also expressed in gliomas [12], but also in some normal structures of the brain [12]. This property limits its interest as a specific tumor antigen for glioblastoma immunotherapy [13]. Interestingly, we have previously found that the *O*-acetylated derivative of GD2—*O*-acetyl-GD2 (OAcGD2)—is not detected in normal brain tissue [13]. However, the distribution of this antigen in GD2-expressing brain tumors remains largely unknown. Here we explored the usefulness of OAcGD2 as a potential tumor antigen for immune-based therapy in patients with malignant glioma. We report that OAcGD2 is specifically expressed in glioblastoma and further demonstrate the *in vitro* and *in vivo* anti-glioma properties of anti-OAcGD2 mAb.

## RESULTS

### Ganglioside OAcGD2 is expressed on human glioblastoma

**Figure 1:**
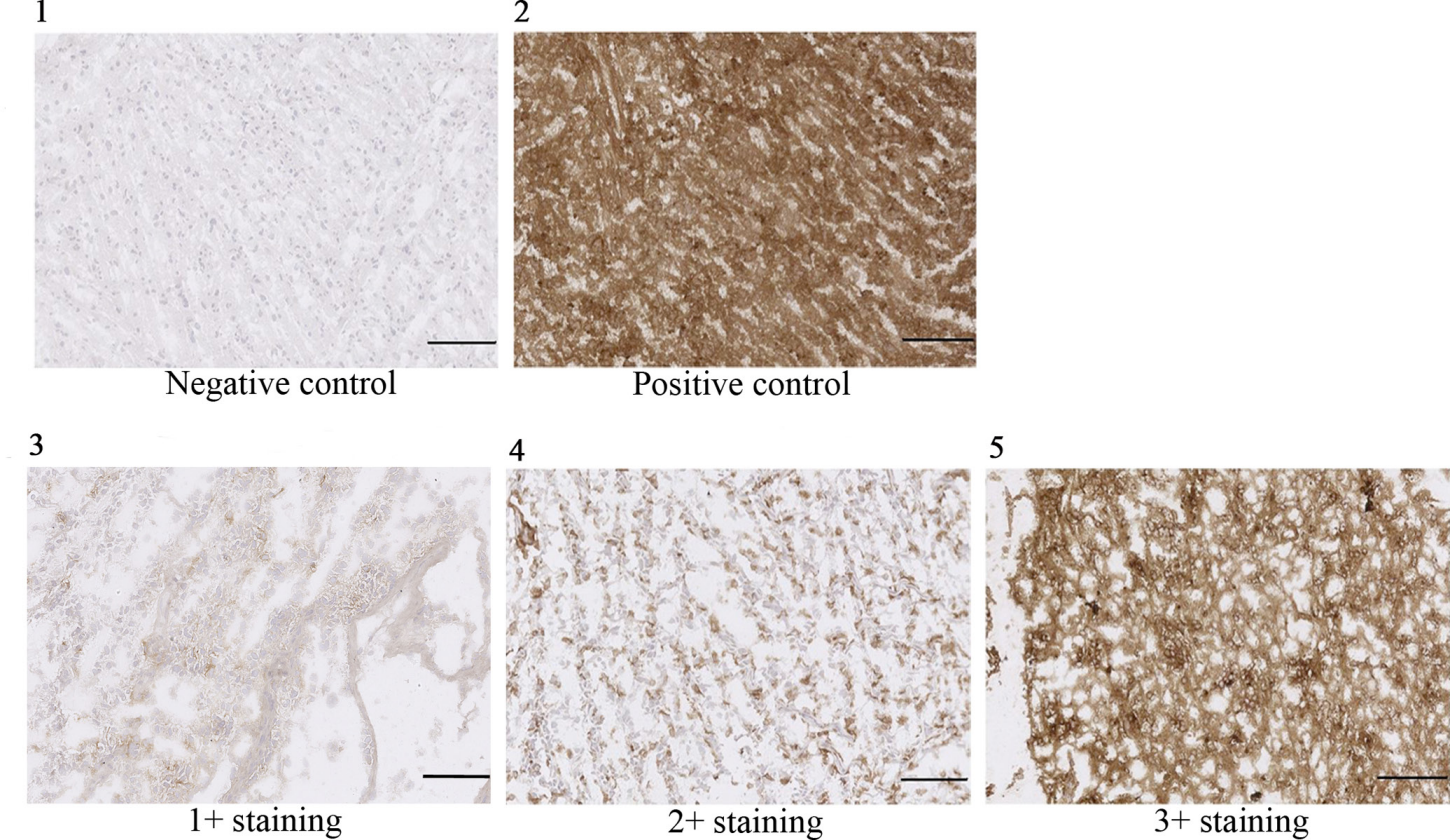
OAcGD2 staining of glioma frozen tissue sections Panel (**1**) is demonstrating a representative picture of negative control (glioma tissue stained with non-specific mAb), panel (**2**) a representative picture of positive control (glioma tissue stained with anti-GD2 14G2a), panel (**3**) a representative picture of a 1+ positive glioma sample, panel (**4**) a representative picture of a 2+ positive glioma sample, and panel (**5**) a representative picture of a 3+ positive glioma sample. Scale bar = 100 μm.

We reported earlier that OAcGD2 expression is absent in brain tissue [13]. Here, we investigated the expression of OAcGD2 in 35 glioblastomas from 35 patients. This panel was obtained from the IRCNA Tumor Bank (Saint-Herblain, France). We performed immunohistochemical staining using 8B6 mAb specific for OAcGD2 on frozen tumor tissue sections. We next graded qualitatively the intensity of OAcGD2 staining compared with positive and negative control samples (Figure 1). Figure 1 shows positive (brown) 8B6 staining in 3 human glioma samples with 3+, 2+ and 1+ immunochemical intensity. We detected positive staining in all 35 glioma tumor samples studied (Table 1, Supplementary Figure S1). Nine tumors (25.7%) demonstrated 1+ immunochemical intensity, 17 tumors (48.6%) demonstrated 2+ intensity, and nine tumors (25.7%) demonstrated 3+ intensity. The data showed no correlation between the OAcGD2-expression level and the age or the sex of the patients (Table 1). These results show that expression of OAcGD2 can serve as a marker of glioblastoma.

**Table 1:** Expression of OAcGD2 in glioblastomas

N°	Sex	Age	Type	8B6 mAb staining^a^
1	M	69	IV	1+
2	F	45	IV	1+
3	M	62	IV	1+
4	F	78	IV	1+
5	M	62	IV	1+
6	M	46	IV	1+
7	M	66	IV	1+
8	F	64	IV	1+
9	F	48	IV	1+
10	F	37	IV	2+
11	M	64	IV	2+
12	M	46	IV	2+
13	M	77	IV	2+
14	M	72	IV	2+
15	F	68	IV	2+
16	F	73	IV	2+
17	M	61	IV	2+
18	M	65	IV	2+
19	M	61	IV	2+
20	F	59	IV	2+
21	F	69	IV	2+
22	F	67	IV	2+
23	F	69	IV	2+
24	M	74	IV	2+
25	F	65	IV	2+
26	M	58	IV	2+
27	M	66	IV	3+
28	M	68	IV	3+
29	F	49	IV	3+
30	M	59	IV	3+
31	F	58	IV	3+
32	F	57	IV	3+
33	F	61	IV	3+
34	F	54	IV	3+
35	M	74	IV	3+

a The staining intensity of the glioma tissues was graded as 0 (negative), 1+ (weak), 2+ (moderate), and 3+ (strong) after immunohistochemical staining with mAb 8B6, as described in the Materials and Methods section.

### Anti-OAcGD2 mAb binds to primary glioma biopsy-derived cells and glioma cell lines

**Figure 2:**
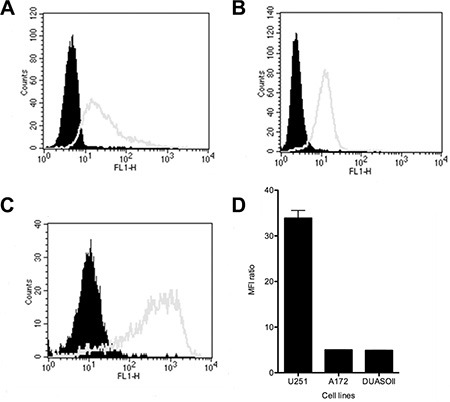
The expression levels of OAcGD2 on the A172 cell line (A), the U251 cell line (B), and on the DUASO II tumor-derived cells (C) were studied by flow cytometry as indicated Tumor cells were stained with mAb 8B6 as described in the Materials and Methods section. The histograms of the cells stained with mAb 8B6 and isotype control are shown in white and black, respectively. (**D**) Comparison binding of mAb 8B6 on DUASO II, A172 and U251 cells. Results are presented as MFI ratios calculated by using geometric mean values of the MFI as indicated in the Materials and Methods section. Data are expressed as mean ± SD (*n* = 3 independent experiments with similar design).

We next characterized the expression levels of OAcGD2 by flow cytometry in 3 glioma- and 9 patient-derived glioma cells. We established these primary GBM cells from patients with glioblastoma undergoing surgery at G. Laennec Nantes University Hospital (Nantes). Figure 2 shows the flow charts of the comparative binding of mAb 8B6 on the patient-derived glioma cells DUASO II, the human U251- and human A172 cell lines. The data obtained with the other studied glioma cells are presented in Supplementary Figure S2 and in Table 2. We found that all studied primary glioma biopsy-derived cells and GBM cell lines expressed OAcGD2 ganglioside. We observed no binding of control antibody to glioma cells, confirming the specificity of mAb 8B6 staining (Figure 2). Analyses of the MFI ratios indicated that the patient-derived glioma cells expressed higher level of OAcGD2 than the GBM cell lines (Figure 2, Table 2).

**Table 2:** *In vitro* anti-tumor properties of mAb 8B6 against GBM cells

Tumor cells	OAcGD2 expression^a^	Direct cytotoxicity^b^	CDC^c^	ADCC^d^
**Tumor-derived GBM cells**				
AMBMA	22.2	13.3 ± 1.4	17.6 ± 1.1	11.9 ± 1.0
BAUJE	52.2	0	0	20.0 ± 0.4
BICYY	12.2	0	0	23.5 ± 1.2
COXCA	24.2	0	0	22.3 ± 1.9
GBMAI	4.6	0	0	26.3 ± 2.1
Glio 5	29.7	8.7 ± 1.2	0	39.5 ± 3.7
GUITH	14.3	11.4 ± 1.0	0	37.8 ± 1.3
HOUHE	9.4	0	0	19.0 ± 0.5
**Cell lines**				
LN18	4.2	10.6 ± 0.9	0	8.6 ± 0.7

a Determined by flow cytometry experiments using an immunofluorescent staining with mAb 8B6, as described in the Materials and Methods section.

b Determined as tumor viability inhibition (%) by MTT assays after 24 hours incubation with mAb 8B6 (50 μg/mL).

c Determined as tumor cell lysis (%) by flow cytometry experiments after incubation for 4 hours with mAb 8B6 (10 μg/mL) with 20% of human serum.

d Determined as tumor cell lysis (%) by flow cytometry experiments after incubation for 24 hours with mAb 8B6 (10 μg/mL) at an E/T ratio of 1/12.5 with 10 μg/ml of mAb 8B6.

### Antibody 8B6 induces tumor cell death of OAcGD2-expressing glioma cells *in vitro*

**Figure 3:**
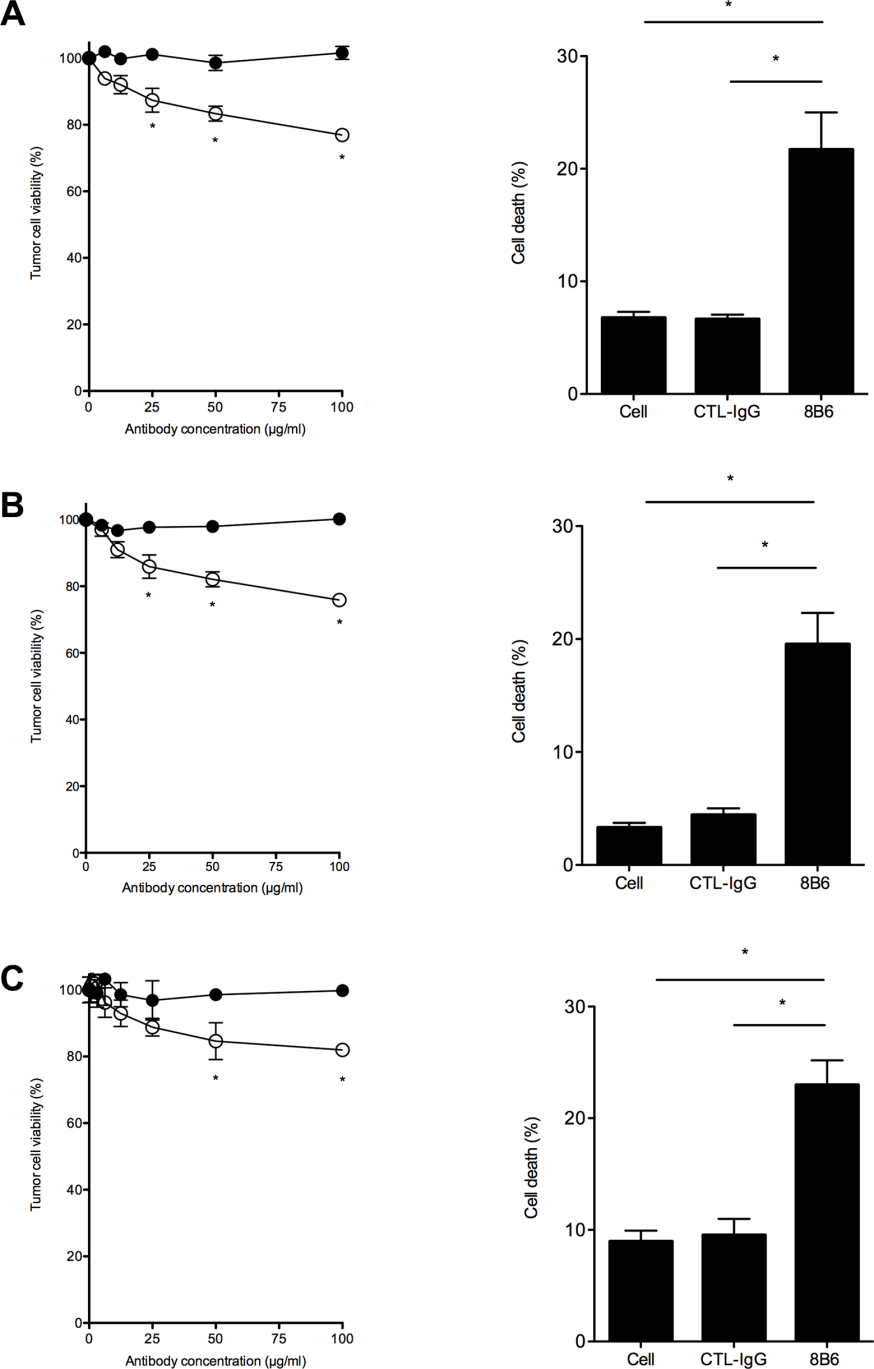
Antibody 8B6 decreases cell viability of OAcGD2-expressing glioblastoma cells Left column panels, U251 (**A**), A172 (**B**), and DUASO II (**C**) cells were treated for 24 hours with various concentrations of mAb 8B6 (○) and a control antibody (●). Viability was assessed as described in the Materials and Methods section by adding the methylthiazole tetrazolium salt during 4 hours (MTT assay). Absorbance was recorded at 570 nm. The data are presented as mean ± SD for three independent experiments, each in quadruplicate (*p < 0.05). Right column panels, cell viability after 24 hours of incubation with mAb 8B6 was evaluated by propidium iodide uptake and flow cytometry analysis. Cytotoxicity is expressed as percentage of propidium iodide-stained cells. Isotype-matched 7H2 antibody was used as a negative control as indicated. Bars represent mean of triplicate measurements; SD are shown.

We next sought to determine whether mAb 8B6 could inhibit glioma cell growth. To assess this question *in vitro*, we first determined the tumor cell viability after mAb 8B6 treatment using a MTT assay (Figure 3, left column panels). We included a control antibody to ensure that the observed results were antigen specific. We observed an inhibition of tumor cell viability induced by mAb 8B6 in seven of the 12 studied GBM cells (58%) (Figure 3, Table 2). In sensitive cells, the cell viability reduction induced by mAb 8B6 was dose- and time-dependent (data not shown). For the U251 cell line, the effect became statistically significant after 24 hours at 25 μg/mL (*p* < 0.05), as compared to control antibody-treated cells (Figure 3A). The highest inhibitory effect was exerted by the antibody concentration of 50 μg/mL. This treatment resulted in a |20% decreased in U251 cell viability (Figure 3A). Similarly, viability of A172 (Figure 3B), DUASO II (Figure 3C), LN18 (Table 2), AMBMA (Table 2), and GUITH (Table 2) cells was also significantly reduced with mAb 8B6 compared to control antibody (*p* < 0.05). We further tested mAb 8B6 ability to induce GBM cell death by staining the tumor cells with propidium iodide followed by flow cytometry analysis. We show in Figure 3, right column panels, the results obtained with mAb 8B6 when the cells were treated with 50 μg/ml for 24 hours. Antibody 8B6 induced cell death in the 3 tested GBM cell types. On the other hand, the control antibody did not affect the cell viability compared to the untreated cells. We also found that mAb 8B6-induced cell death was associated with an increased percentage of annexin V positive cells (Supplementary Figure S3) and the activation of caspase 3 (Supplementary Figure S4) in comparison to control antibody-treated- and untreated-cells. Interestingly, the effects of mAb 8B6 on glioma cell viability were partially blocked by pre- treatment of the tumor cells with the pan-caspase inhibitor zVAD- fmk (Supplementary Figure S4). This suggests that the observed effects were, at least in part, caspase-dependent. Overall, these results show that mAb 8B6 induces GBM cell death independently of immunological mechanisms *via* the caspase-3-dependent pathway and some independent pathways.

### Antibody 8B6 induces immune effector activity against OAcGD2-expression glioma cells

**Figure 4:**
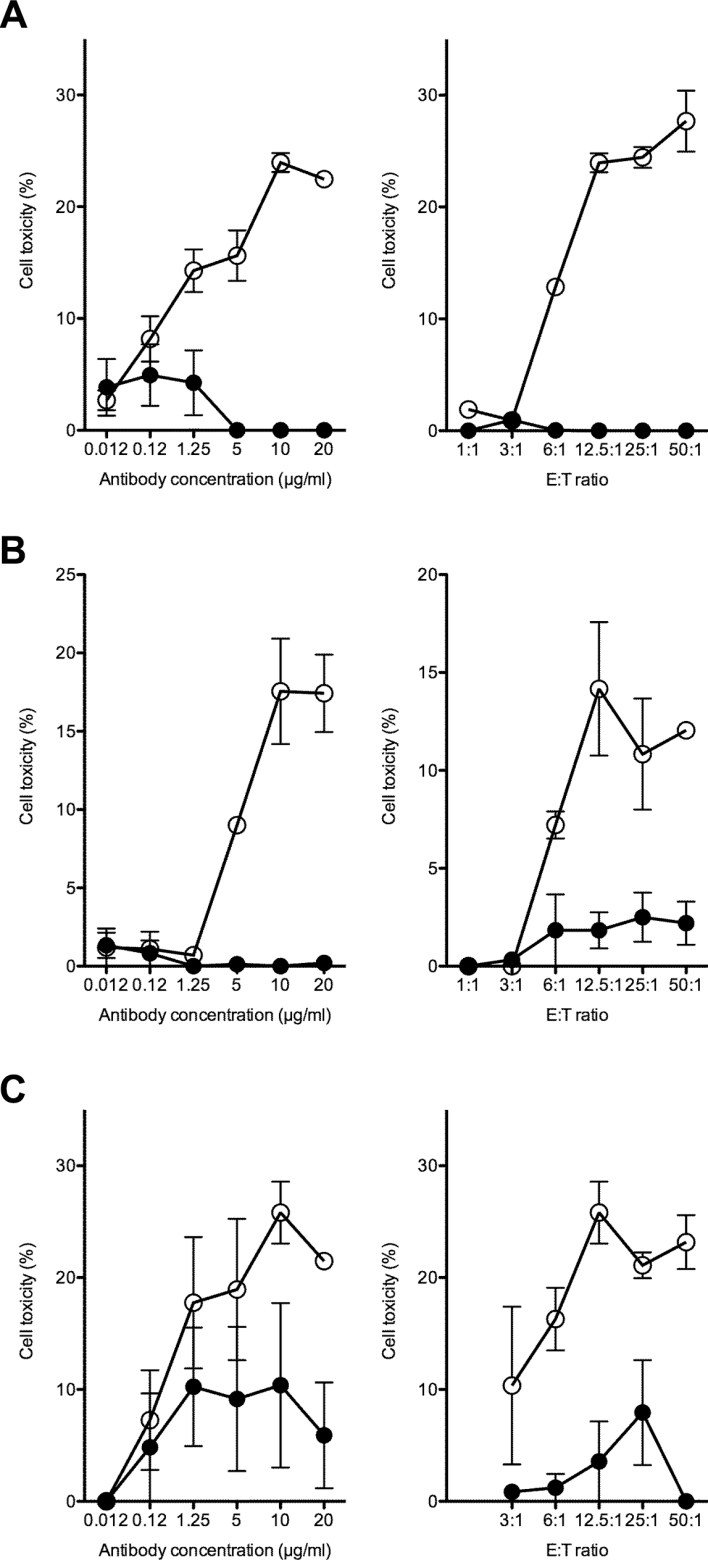
ADCC activity of mAb 8B6 (○) was determined against the OAcGD2-positive glioblastoma cells U251 (A), A172 (B) and DUASO II (C) Left columns, effect of varying antibody concentration at effector to target cell ratio of 12.5 to 1. Right columns, effect of varying effector to target ratio at antibody concentration of 10 μg/ml. The results were compared to the effect of equal amounts of the isotype-matched control IgG (●). Results are presented as mean ± SD of triplicate sample in a representative experiment. Similar results were obtained in three independent experiments.

Immune effector activity is an important mechanism of antibody against cancer cells and, in particular, antibody-dependant cell cytotoxicity (ADCC) has been implicated in the clinical efficacy of anti-ganglioside antibody [14, 15]. Thus, we studied the effect of mAb 8B6 on ADCC activity against the OAcGD2-expressing glioma biopsy-derived tumor lines. To this end, glioma cells were labeled with the PKH-26 membrane dye, and incubated with various concentrations of mAb 8B6. The NK-92-RFcγIII+ cells were used as effector cells. After incubation, cell death within the PKH-26^+^ target cell population was detected by the addition of the viability probe TP3. We observed ADCC with mAb 8B6 against the U251, the A172 and the DUASO tumor cells (Figure 4). We found a correlation between ADCC activity and both the concentration of antibody and the E/T ratio (Figure 4). Specific lysis achieved maximum values in the 3 studied cell types at an E/T ratio of 1/12.5 with 10 μg/ml mAb 8B6. We demonstrated the specific lysis by comparing the ADCC results of mAb 8B6 with the control antibody, which showed only background lysis. We found similar results with the LN18 cell line and the 8 other studied patient-derived GBM cells (Table 2).

**Figure 5:**
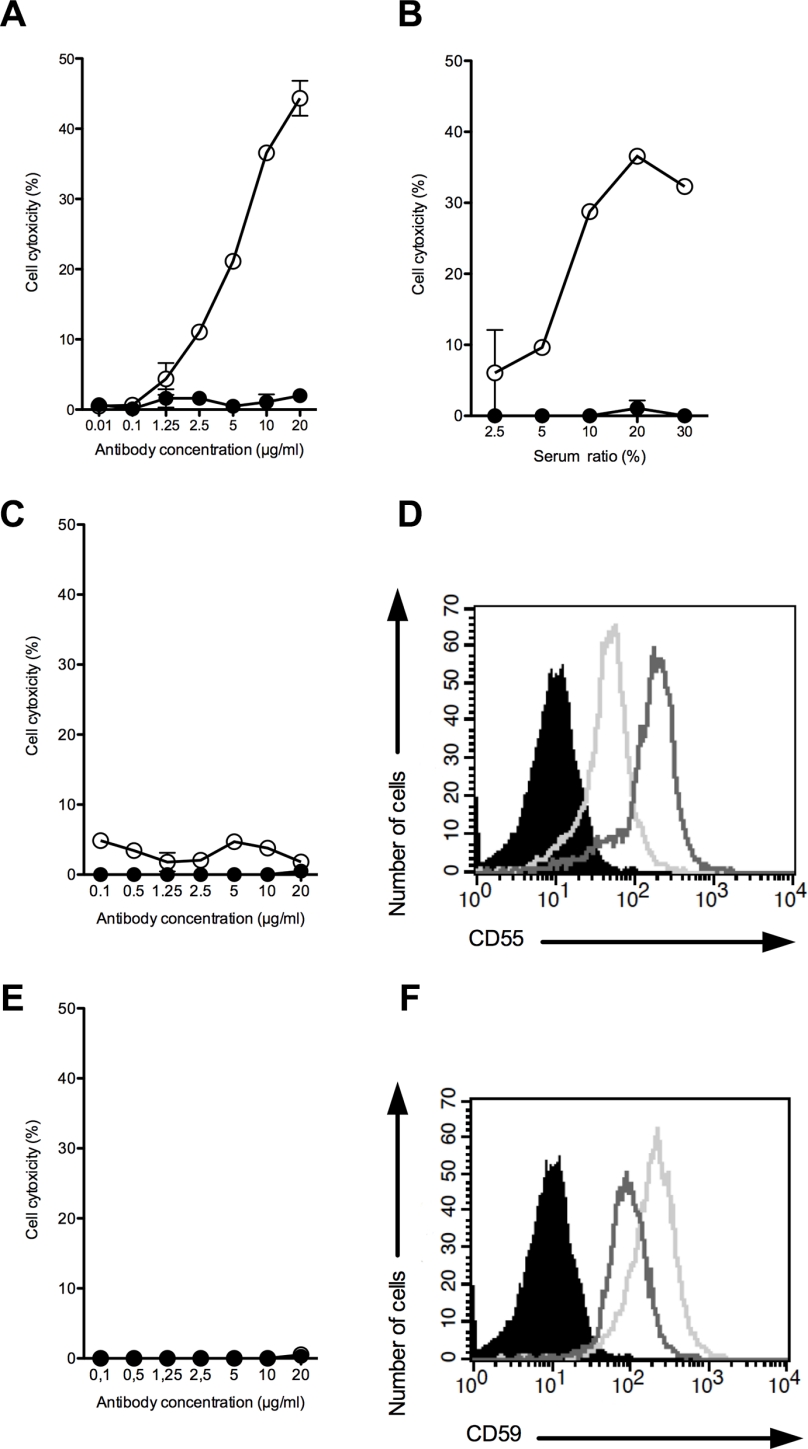
CDC activity of mAb 8B6 (○) at varying antibody concentrations against DUASO II cells (A, B), U251 cells (C), and A172 cells (E) CDC activity of mAb 8B6 (○) (10 μg/ml) was assessed against the OAcGD2 positive DUASO II tumor-derived cells at varying serum ratios. The results were compared to the effect of equal amounts of control antibody used as a negative control (●). Results are presented as mean ± SD. The expression of the inhibitors of complement activation, CD55 (**D**) and CD59 (**F**), on the CDC-resistant glioblastoma cell lines, U251 (bold line) and A172 (grey line), was analyzed by flow cytometry. The cells stained with the isotype control antibody are shown in black.

To this end, we incubated the OAcGD2-expressing target cells with mAb 8B6 in the presence of diluted human serum, which provided complement. Cell death was assessed by the addition of the viability probe propidium iodide. We found that mAb 8B6 induced CDC towards the DUASO tumor-derived cells (Figure 5). We observed a correlation between cytotoxicity and both the concentration of antibody (panel A) and the serum ratio (panel B). Specific lysis achieved maximum values of 36.6 ± 0.2% with mAb 8B6, at 10 μg/ml and serum ratio of 10%. We demonstrated the specific lysis by comparing the CDC results of mAb 8B6 with the non-specific control antibody, which showed only background lysis. We obtained similar results with the AMBMA primary cells (Table 2). We found that the U251 (Figure 5C) and the A172 cell lines (Figure 5E) were resistant to CDC induced by mAb 8B6. These two cell lines expressed the inhibitors of complement activation CD55 (Figure 5D) and CD59 (Figure 5F). The BAUJE, COXCA, GBMAI, Glio 5 and HOUHE GBM primary cells were also resistant to CDC induced by mAb 8B6 (Table 2). Taken all together, these data suggest that ADCC is the main mAb 8B6 GBM killing mechanism.

### Antibody 8B6 to OAcGD2 inhibits glioma xenograft growth

**Figure 6:**
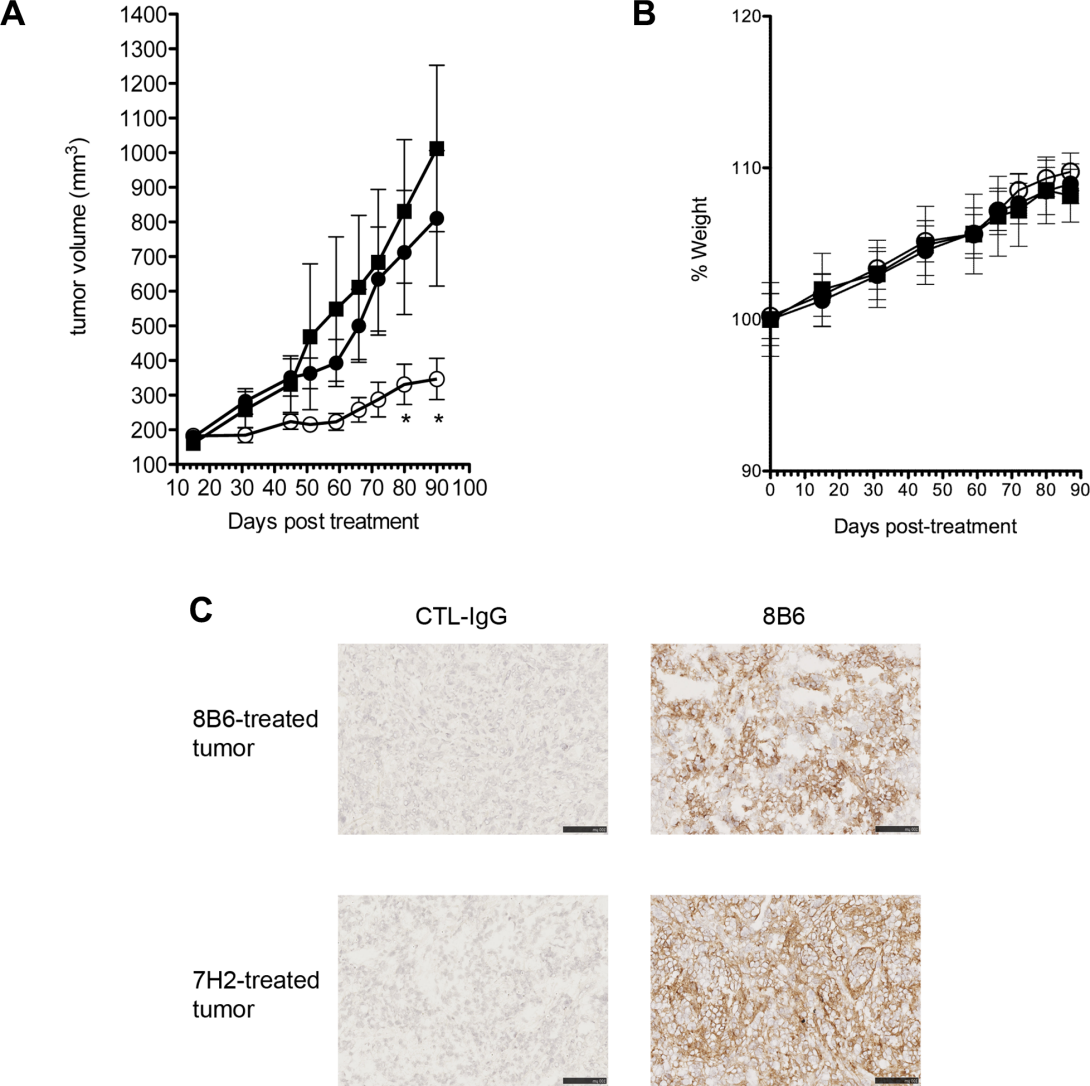
(**A**) Anti-OAcGD2 mAb 8B6 inhibits glioblastoma tumor growth *in vivo*. Mice (*n* = 12) with established subcutaneous U251 human glioblastoma xenografts (average volume |150 mm^3^) were treated with either PBS (■), control mAb (●), or mAb 8B6 (○). Tumor growth was monitored and mice were euthanized for ethical consideration once the tumor volume exceeded 1000 mm^3^, which was therefore considered the end point for each individual mouse. (**B**) Mean weight for each treatment group (■, PBS; ●, control antibody; ○, 8B6 antibody). Mean weight of group of mice (*n* = 12) at day 0 was defined as 100% weight. Results are presented as mean ± SD. (**C**) Tumors were collected on day 90 after mAb 8B6 immunotherapy perfusion. Strong immunostaining with biotinylated-8B6 mAb was observed on U251 tumor cells in the mAb 8B6 treatment regimens. The control antibody was used as a negative control, as indicated. Three U251 tumor xenografts from 3 different mice in the 8B6 and the control antibody experimental group were tested with the same result respectively. Scale bar = 50 μm.

We next investigated the effect of anti-OAcGD2 mAb 8B6 on glioblastoma growth. We used a subcutaneous U251 human glioblastoma xenograft model. When tumors reached 150 mm^3^, we treated the mice with a single i.v. injection of 250 μg mAb 8B6 and followed tumor growth. We also included a control antibody-treated group to demonstrate the specificity of the mAb 8B6-therapy. As shown in Figure 6A, mAb 8B6 significantly inhibited the tumor growth. The tumor volume at day 90 after antibody injection, volumes averaged 1023 ± 240 mm^3^, 810 ± 195 mm^3^, and 346 ± 59 mm^3^ when the mice were treated respectively with vehicle (PBS group), with CTL-IgG (control group) or mAb 8B6. In this context, mAb 8B6 injection resulted in a significant increase of the U251 tumor doubling time, compared to both PBS- and CTL-IgG-treated group (Supplementary Figure S5, *p* < 0.05). The antibody treatment did not induce any body weight loss during the experiment (Figure 6B). Taking into consideration that specific immunotherapy can result in antigen-negative tumor recurrence [16], we next analyzed OAcGD2 expression in the recurrent U251 xenograft in the 8B6-mice treated group. We found that biotinylated-8B6 mAb stained strongly U251-tumor sections similarly in mice treated with either mAb 8B6 or control antibody (Figure 6C). In contrast, we could not detect any labeling with the biotinylated-control antibody (Figure 6C). Taken together these results show that anti-OAcGD2 immunotherapy inhibits OAcGD2-expressing glioblastoma tumor growth without down-regulating OAcGD2 expression on tumor cells.

## DISCUSSION

Here, we exploited the availability of a panel of human glioma surgical specimens to characterize their OAcGD2 status. We observed OAcGD2 positivity in the 35 patients: 9 patients (26%) displayed 1+ tumors, 17 patients (49%) 2+ tumors, and 9 (26%) 3+ tumors. We also found three cell lines and nine primary glioma tumor cells expressing OAcGD2. Important in the consideration of OAcGD2 as a potential molecular antigen for immunotherapy, we reported earlier that no comparable immune reactivity could be observed in normal brain or in peripheral nerves [13]. Thus, the immune reactivity throughout the entire studied panel of glioma biopsies for mAb 8B6 supports the clinical use of therapeutic antibody specific for OAcGD2. To the best of our knowledge this is the first investigation describing the expression of OAcGD2 in tumor surgical samples [17].

To confirm that OAcGD2 ganglioside is a suitable antigen for glioblastoma immunotherapy, we next studied the anti-glioblastoma activity of the mAb 8B6 specific for OAcGD2 ganglioside. Our results showed that glioblastoma cells expressing OAcGD2 can be killed by mAb 8B6 by three different mechanisms *in vitro*: (1) induction of a non-immunologic direct cytotoxicity involving at least caspase-3, (2) induction of antibody-dependent cell cytotoxicity, and (3) induction of complement cellular cytotoxicity. Here, we found that most of the studied glioblastoma cell lines and glioblastoma tumor-derived cells were resistant to CDC in our *in vitro* experiments. We explained the lack of effect to the overexpression in these cells of the two complement inhibitors CD55 and CD59, as shown in our flow cytometry analyses. Surprisingly, the non-immunologic direct cytotoxicity, which we described earlier in neuroblastoma cells [18], occurred in only 58% of the studied glioblastoma cellular models. These data warrant further studies to define the molecular mechanisms that confer tumor cell resistance to apoptosis induced by anti-OAcGD2 mAbs. We also demonstrated that mAb 8B6 induces ADCC against glioblastoma cells. This capacity should be considered as important feature goal for anti-OAcGD2 immunotherapy. For example, the development of engineered cytotoxic T-cells expressing a chimeric antigen receptor specific for OAcGD2 may be a better choice to strengthen glioblastoma immunotherapy than a stand alone antibody therapy [19]. Due to its exquisite tumor-specificity [13], OAcGD2 could also represent an ideal antigen for chimeric antigen receptor-based cellular therapies.

Finally, to support our findings *in vitro*, we evaluated the efficacy of mAb 8B6 in inhibiting the growth of established subcutaneous U251 human glioblastoma xenografts. Similar to our *in vitro* data, a therapeutic effect was obtained *in vivo*. The anti-OAcGD2 mAb 8B6 was able to delay tumor growth of animals with subcutaneous established U251 glioma xenograft. Taking into account that therapeutic antibody attack can select antigen-negative clones with subsequent high-risk of recurrence of antigen-negative tumors, we assessed OAcGD2 expression in the recurrent tumor of mAb 8B6 treated mice 90 days after antibody injection. Loss of glioblastoma antigen expression upon specific immunotherapy was recently illustrated in an immunization study against EGFRvIII [8]. Patients with initial response ultimately showed recurrence of EGFRvIII-negative tumor [8]. Here, we found that OAcGD2 expression was persistent in the U251 xenografts post-immunotherapy. This observation is consistent with an earlier study concerning the persistence of the neuroblastoma-associated ganglioside GD2 antigen after anti-GD2 therapeutic antibody therapy [20]. This stable expression of OAcGD2 on GBM tumors is an important prerequisite for OAcGD2-directed immunotherapies. Hence, as a specific glioblastoma antigen, OAcGD2 has many attractive properties, including high tumor density, homologous tumor cell expression, and expression persistence post-immunotherapy.

In conclusion, we have reported that OAcGD2 ganglioside is expressed at high levels in glioblastoma tumor and that mAb 8B6 specific for OAcGD2 efficiently inhibits glioblastoma growth. These results warrant the development of antibody constructs for application in immunotherapy of GBM.

## MATERIALS AND METHODS

### Cell lines

Human glioblastoma (GBM) cell lines (U251, LN18, and A172) were obtained from the American Tissue Culture Collection (ATCC, USA). Cell lines were used for less than 6 months after resuscitation and were routinely tested for *Mycoplasma* by PCR. We did not conduct any genotypic authentication. U251 and A172 cell lines were cultured in DMEM 4, 5 g/l glucose, supplemented with 10% fetal calf serum (FCS) and L-glutamine. LN18 cell line was cultured in DMEM 4, 5 g/l glucose, supplemented with 5% FCS and L-glutamine. All cell lines were cultured with 100 units/ml penicillin and streptomycin. All culture reagents were obtained from Gibco Life Technologies (Carlsbad, CA, USA). They were maintained in culture at 37°C with 5% CO_2_.

### Primary glioma biopsy-derived cells

Tumor specimens were collected from patients with a histologic diagnosis of GBM (WHO Grade IV astrocytoma). Confirmation of tumor diagnosis and grading was performed by neuro-pathologists at Nantes University Hospital (Nantes, France). Tumors were harvested at the time of surgical resection and immediately put into culture after dissociation of the tumors using the gentleMACs Dissociator (Miltenyi) according to the manufacturer's instructions. All specimen collection and analysis were performed in accordance with the Institutional Review Board-approved protocol and all patients or their guardians provided written informed consent (*Comité de Protection des Personnes Ouest IV, protocol # DC-2012-1555*). The cells were maintained in an atmosphere of 5% CO_2_ and 95% humidity in defined medium (DMEM/Ham F12 containing 2 mM L-glutamine, 100 U/mL penicillin, 100 μg/mL streptomycin, B27 supplement, N2 supplement, 2 μg/ml heparin, 40 ng/ml ß-FGF and 40 ng/ml EGF) to form neurospheres. They were used at early passage. We conducted genotypic authentication using the CGH Array (ThermoFisher Scientific, Waltham, MA, USA). All the cell cultures were tested for *Mycoplasma* contamination by PCR before use.

### Antibodies

Mouse IgG2a mAb 14G2a (γ2a, kappa) specific for GD2 was purchased from BD Biosciences (Franklin Lakes, NJ). Mouse IgG2a mAb 7H2, a gift from Dr. J. Portoukalian (Department of Dermatology, Edouard Herriot Hospital, University of Lyon, France) specific for *O*-acetyl-GD3, was used as a negative control. Mouse IgG2a anti-OAcGD2 mAb 8B6 was constructed by joining the complementary deoxyribonucleic acid for the variable region of the parental murine IgG3 antibody 8B6 [9] with the mouse constant regions of the γ2a heavy chain and the kappa light chain. Appropriate light and heavy expression vectors were co-transfected into chinese hamster ovary (CHO-S) cells (Life Technologies). The resulting antibody was affinity-purified from culture supernatant using the Hitrap rProtein A FF column (GE Healthcare Bios-Sciences, Uppsala, Sweden). The protein A affinity chromatography step was followed by anion-exchange chromatography on Sepharose Q for endotoxin removal. The purity of mAb preparations was verified by SDS-PAGE and size exclusion HPLC analyses. Endotoxin quantitation was evaluated using the LAL kinetic chromogenic assay (Lonza, Basel, Switzerland).

### Immunohistochemistry on human glioblastoma samples

Glioblastoma tissues were provided by the Tumor Bank of the “*Institut Régional du Cancer de Nantes Atlantique”* (Nantes, France). Samples were obtained from patients at the time of surgery. All samples had **≤** 50% necrosis to be able to accurately assess OAcGD2 expression across specimens. All specimen collection and analysis were performed in accordance with the Institutional Review Board-approved protocol and all patients or their guardians provided written informed consent (*Comité de Protection des Personnes Ouest IV, protocol # DC-2012-1555*). Frozen tissue sections of 10 μm thickness were treated with ice-cold acetone, rehydrated with PBS and blocked with the Dako Real^™^ antibody diluent reagent (Dako, Glostrup, Denmark). Antibody 8B6 was added onto the sections at a final concentration of 10 μg/ml for 1 hour. After washing, the bound mAb was detected by incubation of anti-mouse labeled polymer-HRP (Dako). DAB was used as HRP substrate and sections were counterstained with hematoxylin before immune cytological evaluation. Anti-GD2 mAb 14G2a was used as a positive control and mAb 7H2 as a negative control. Slides were imaged with a Nanozoomer (Hamamatsu, Hamamatsu City, Japan). Images were stored as TIFF files with Adobe Photoshop. Staining was graded positive or negative according to the presence or the absence of immune reactivity, respectively. The intensity and location of tissue staining were assessed by a comparison with the positive and negative controls. Tissue was assessed and graded by 2 independent observers and assessments were re-reviewed by an anapathologist.

For detection of OAcGD2 in the U251 xenografts, we use biotinylated mAb 8B6. The mAb 8B6 was biotinylated using the EZ-Link Sulfo-NHS-LC-Biotinylation kit (Pierce, Rockford, IL, USA) according to the manufacturer's instructions. Ten micrometer-sections of the U251 xenografts were blocked with the Dako Real^™^ antibody diluent reagent as described above (Dako, Glostrup, Denmark). We then added 8B6-biotinylated mAb onto the sections at a final concentration of 10 μg/ml for 1 hour. After rinsing, the bound mAb was detected by incubation with Streptavidin-HRP. DAB was used as HRP substrate and sections were counterstained with hematoxylin before immune cytological evaluation. Biotinylated-7H2 mAb was used as a negative control. Slides were imaged and pictures were stored as described above.

### Analysis of cell-surface expression of OAcGD2 by flow cytometry

Analysis of cell surface OAcGD2-expression on tumor cells was performed by indirect immunofluorescence measured by flow cytometry. Briefly, cells were incubated with either mAb 8B6 or 7H2 at 10 μg/mL for 60 min at 4°C. Antibody binding was detected by incubation with a fluorescein isothiocyanate-labeled F(ab')_2_ fragment of goat anti-mouse IgG (Jackson Immunoresearch, Soham, UK) for 60 min at 4°C. Cell fluorescence was analyzed using a FACSCalibur flow cytometer (BD Biosciences, San Jose, CA, USA) and CellQuestPro software (BD Biosciences). Relative fluorescence intensities of 10,000 cells were recorded as single-parameter histograms (log scale, 1024 channels, and 4 decades) and mean fluorescence intensity (MFI) was calculated for each histogram. Results were expressed as the MFI ratio calculated by dividing the flow cytometric MFI value of cells stained with antigen-specific mAb by the MFI value for the same cells stained with isotype-matched control antibody. This approach allows for comparison of multiple test samples between different groups.

### Cell growth inhibition

Cell viability was measured using the MTT assay [21]. Cells (1 × 10^4^) were suspended in 100 μL and incubated for 24 hours at 37°C, 5% CO_2_. Monoclonal antibodies were diluted in 50 μL specific medium and added to 96-well microplates (BD, Franklin Lakes, NJ, USA) to give a final concentration of 40, 20, 10, 5, 2.5, and 1.25 μg/mL. After incubation for 24 hours at 37°C, 5% CO_2_, 10 μL methylthiazole tetrazolium (MTT) stock solution (5 mg/mL, Sigma Aldrich, Saint Louis, MO, USA) was added to each well and the plates were incubated at 37°C for 4 hours. Then, 100 μL 10% SDS were added and the plates were incubated for overnight at 37°C for color development. Optical density was recorded at 570 nm and at 650 nm on a Multiskan reader (Thermo Electron, Walthman, MA, USA).

### Induction of cell death

Cells were seeded in 48-well culture plates and incubated with the antibodies (50 μg/ml) for 24 hours. Cells were washed, resuspended in PBS with propidium iodide (10 μg/ml; Sigma Aldrich) and analyzed by flow cytometry. Propidium iodide-stained cells were considered as dead cells. A quantitative measurement of dead cells was done by propidium iodide labeling histogram.

### Complement dependent cytotoxicity (CDC)

Aliquots of tumor cells (2 × 10^4^) were incubated with 80 μL antibody solution at various concentration in the presence of 20 μl human serum as complement source for 2 hours at 37°C. Cytotoxicity was determined within the tumor cell population after addition of the viability probe propidium iodide (PI; 10 μg/ml) using a FACSCalibur flow cytometer (BD Biosciences, San Jose, CA, USA) and CellQuestPro software (BD Biosciences). The percentage of specific lysis was calculated as: (% non-viable PI^+^ 8B6-incubated tumor cells) – (% non-viable PI^+^ control antibody-incubated tumor cells).

### Antibody-dependent cell cytotoxicity (ADCC)

An ADCC assay was performed as reported previously [22]. Tumor cells were labeled with membrane dye PKH-26 (Sigma Aldrich, St. Louis, MO, USA) according to the manufacturer's instructions. Aliquots of the labeled cells (1 × 10^4^ cells/100 μl) were incubated with 50 μL antibodies in 96-well plates. The human NK-92-RFcγIII+ cells were used as effector cells [23]. 50 μL NK-92-RFcγIII+ cells at the indicated effector-to-target ratio (E/T) were added to the tumor cells and incubated for 24 hours at 37°C. Cell death within the PKH-26^+^ target cell population was then assessed by the addition of TO-PRO-3 iodide (TP3) (Life Technologies, Grand Island, NY, USA). The double-positive TP3^+^ PKH26^+^ dead target cell population was detected by flow cytometry analysis using a FACSCalibur flow cytometry (BD Biosciences) and CellQuestPro software (BD Biosciences). The percentage of specific lysis was calculated as 100× (non-viable double-positive target cells)/(non-viable double-positive target cells + viable PKH26^+^ target cells). The lysis of the NK-sensitive mouse T cell lymphoma YAC-1 was used as an indicator of NK-92-RFcγIII+ activity [24].

### Glioblastoma xenograft mouse model

Subcutaneous glioblastoma-bearing mice were obtained by s.c. flank injection of 5 × 10^6^ U251 cells in 8-week old female BALB/c nude mice obtained from Charles River Laboratories (Bois des Oncins, France). Subcutaneous tumor growth was measured at indicated days after tumor implantation using the formula [volume mm^3^ = (length) × (width)^2^ × 0.5]. Antibody (250 μg, one injection) was given i.v. when the tumor volume had reached |0.1 cm^3^. For ethical considerations, mice had to be euthanized once tumor volume exceeded 1,000 mm^3^, which was considered the end point for each individual mouse. The mean tumor doubling time was calculated in each experimental group according to Schwartz, 1961 [25]. This study was carried out in strict accordance with the recommendations in the Guide for the Care and Use of Laboratory Animals of the French Department of Agriculture (agreement number 44–278). The protocol was approved by the Committee on the *Ethics of Animal Experiments of the Région Pays de la Loire* (Permit Number: B4459).

### Statistical analysis

Statistical analysis was performed using Prism software (GraphPad Prism Software, La Jolla, CA, USA). Results in the *in vitro* experiments were analyzed with the unpaired *t*-test and are given as mean ± SD. For *in vivo* experiments, results from mAb-treated group were compared with PBS- and CTL-IgG-treated group using the unpaired *t*-testand are given as mean ± SEM. A value *p* < 0.05 was considered to be significant.

## SUPPLEMENTARY FIGURES AND TABLES


