# Supplementary material for: Targeting and killing glioblastoma with monoclonal antibody to O-acetyl GD2 ganglioside
Source: Oncotarget. 2016 May 9;7(27):41172–85. doi: 10.18632/oncotarget.9226 (PMC5173050; doi:10.18632/oncotarget.9226)
Supplement: Supplementary file 1 [file oncotarget-07-41172-s001.pdf]

Targeting and killing glioblastoma with monoclonal antibody to *O*-acetyl GD2 ganglioside

Supplementary Materials

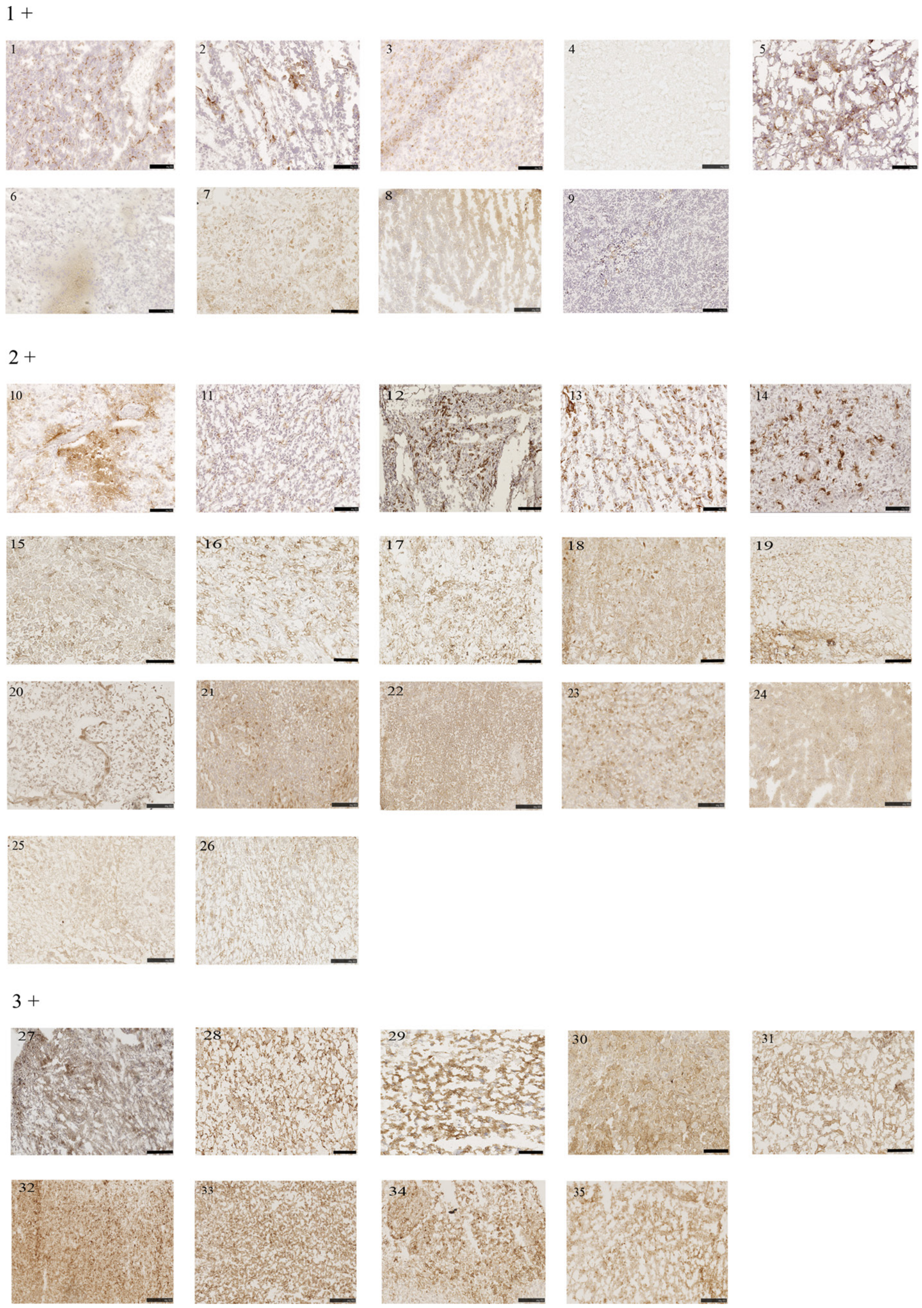

Supplementary Figure S1: Representative examples of OAcGD2 expression on human glioma tumors are summarized in Table 1. OAcGD2 was detected by an immunoperoxydase assay performed with mAb 8B6 as described in the Materials and Methods section. Thirty five Grade IV glioma samples were analyzed. Antibody 8B6 showed moderate (1+) to strong (3+) positive staining with all gliomas. Scale bar = 100 µm.

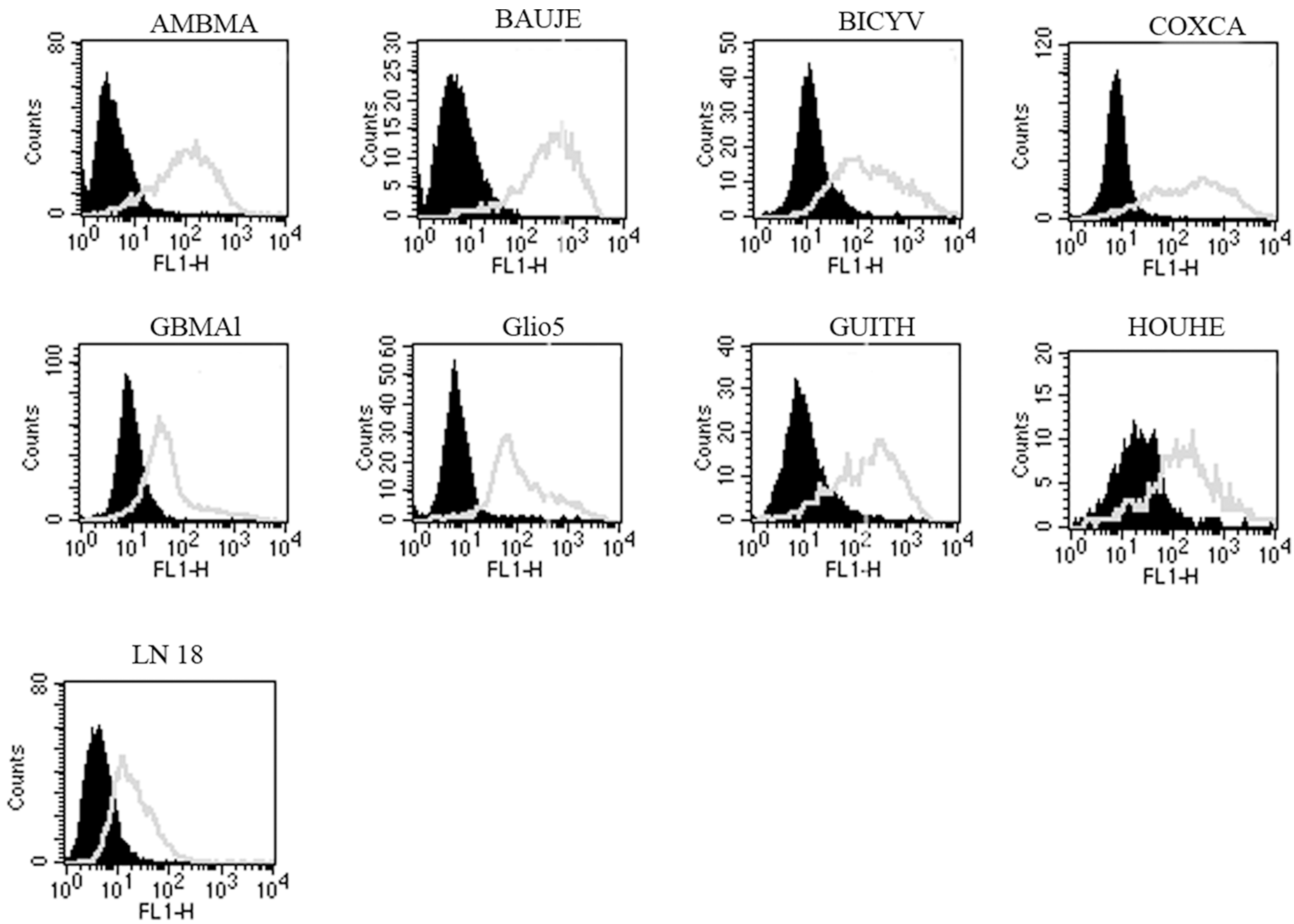

Supplementary Figure S2: OAcGD2 distribution in GBM cell lines and GBM tumor-derived cells. GBM cells were stained with mAb 8B6 and the staining intensity was analyzed with flow cytometry as described in the Materials and Methods section. The histograms of the cells stained with mAb 8B6 and isotype control are shown in white and black, respectively.

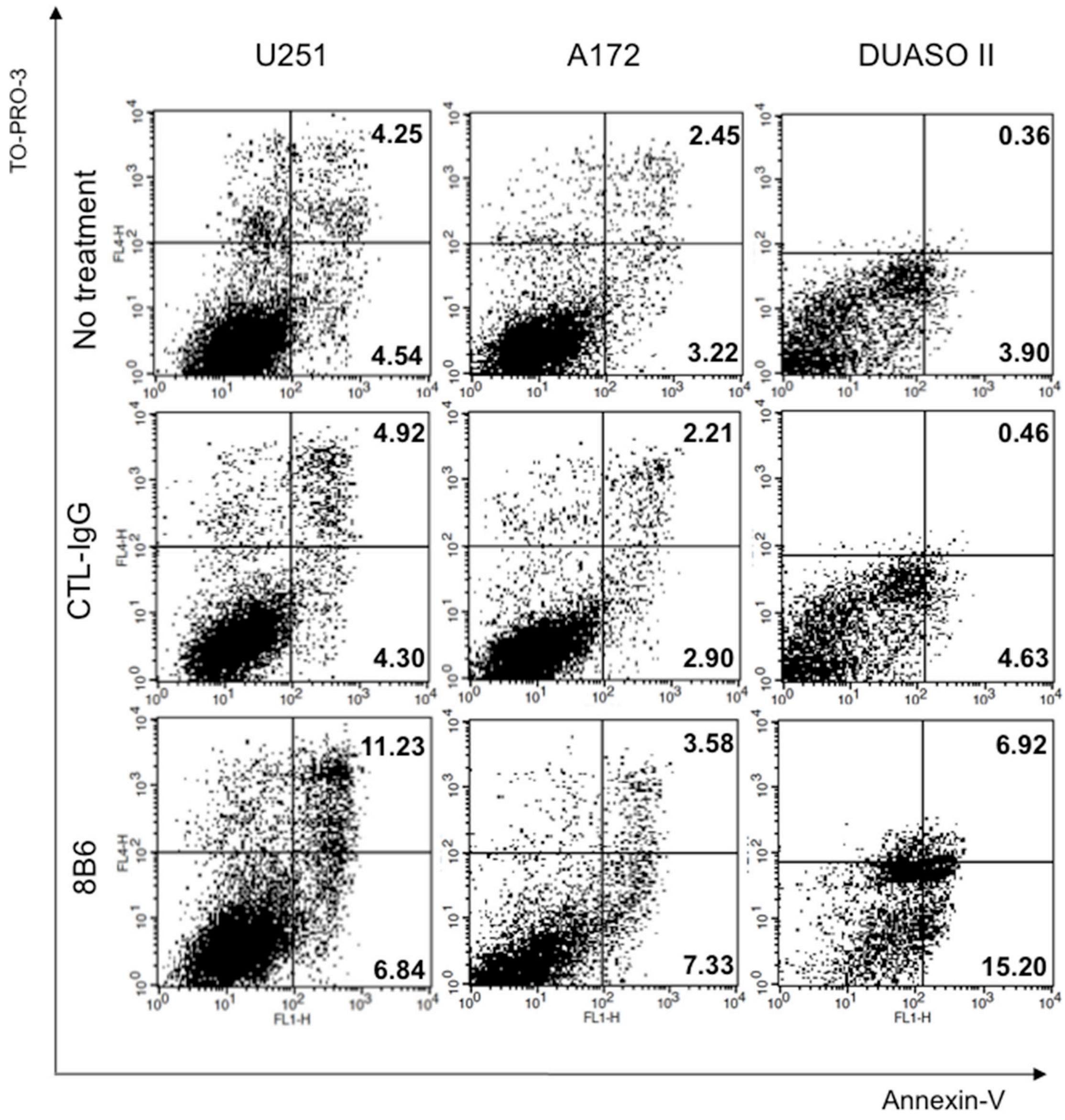

Supplementary Figure S3: Apoptosis detection in mAb 8B6-treated glioblastoma cells. Apoptosis in either U251, A172 or DUASO II cells was quantified using annexin V-FITC/TO-PRO-3 staining followed by flow cytometry analyses after 24 hours incubation with mAb 8B6 (50 µg/ml). The numbers show the percentages of FITC+, TO-PRO-3- early apoptotic cells (lower right quadrant), and FITC+, PI+ late apoptotic or necrotic cells (upper right quadrant). An increase in the percentage of GBM cells exhibiting positivity for annexin V/FITC staining can be observed after treatment with mAb 8B6 (upper and lower right quadrants) compared to control groups. Similar results were obtained at least in three experiments.

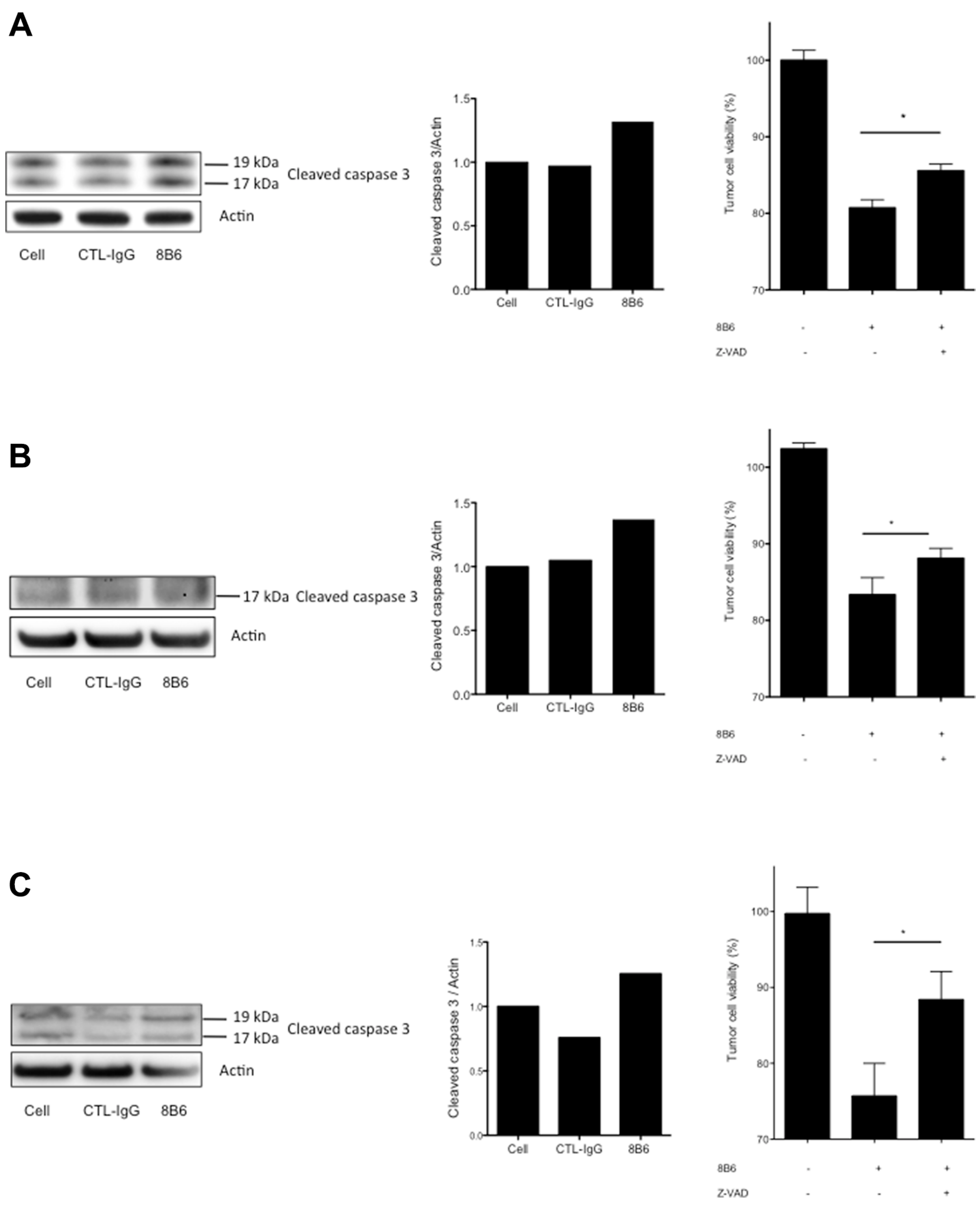

Supplementary Figure S4: Caspase-3 cleavage was seen in either U251 (A), A172 (B), and DUASO II (C) cells exposed to mAb 8B6 for 24 hours. Caspase-3 cleavage was detected by Western blotting with 5A1E mAb. ß-actin was used as a loading control. The left panels show representative images of immunoblots of cleaved-caspase-3 while the Western blot quantification analysis showing the cleaved-caspase-3 band intensity values expressed as fold of ß-actin are presented in the middle panels. Elevated level of cleaved-caspase-3 was seen in all mAb 8B6-treated GBM cells compared to the controls. These observations suggest that mAb 8B6 induced apoptosis in GBM cells. U251 (A) and DUASO II (C) cells contained both p17 and p19 cleaved-caspase-3 subunits. In A172 cells (B), the p17 subunit was detected. Similar results were obtained at least in three experiments. The left panels show that the pan-caspase inhibitor zVAD-fmk partially blocked the cell death induced by mAb 8B6. This suggests that mAb 8B6-induced cell death proceeds by caspase-3-dependent and -independent pathways. Tumor cells were incubated with mAb 8B6 (50 µg/mL) with the presence or absence of zVAD-fmk caspase inhibitor (40 µM), as indicated. After 48 hours incubation, cell viability was determined by MTT assay. Data presented as mean ± SD of triplicate samples in a representative experiment. Similar results were obtained in three independent experiments. $*p < 0.05$.

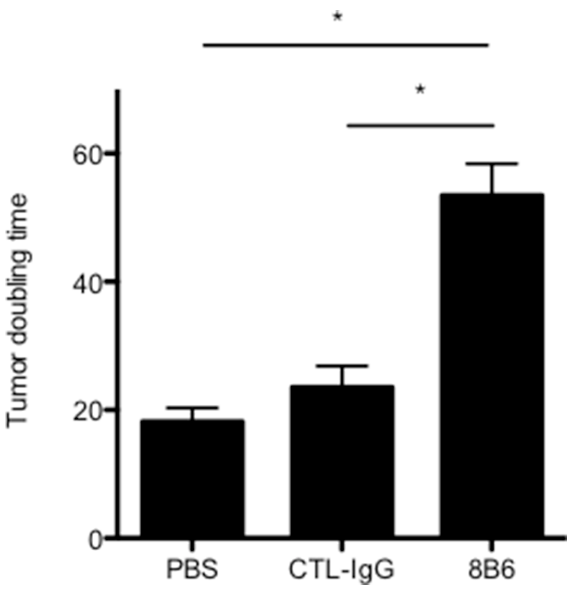

Supplementary Figure S5: Human U251 glioblastoma tumor doubling time observed in the mice depicted in Figure 6. Antibody 8B6 treatment resulted in a reduction of the U251 tumor doubling time compared to either vehicle- or CTL-Ig-treated groups. Data were expressed as mean ± SEM. $^*p < 0.05$.

MATERIALS AND METHODS

Apoptosis induction by mAb 8B6

Cells (1×10^5 cells) were plated in 6-well plates in specific medium during 24 hours at 37°C, 5% CO_2, and then treated for 24 hours at 37°C with either 50 µg/ mL of 8B6 or control mAb, or left untreated. After antibody incubation, cells were washed and resuspended in 100 mL 1X binding buffer (BD pharmingen™, BD Biosciences). Then, cells were incubated with annexin V-FITC (BD pharmingen™, BD Biosciences) and TO-PRO-3 iodide for 15 min. Cell death and undergoing apoptosis were identified by FACSCalibur flow cytometer (BD Biosciences, San Jose, CA, USA) using Cell Quest software. Each assay was done in triplicate. Apoptotic cells were analyzed on annexin V (FL1) histograms and compared to TO-PRO-3 positive and negative cell populations (FL2).

In some experiments, tumor cells were incubated with the OAcGD2-specific mAb (50 µg/mL) in the absence or presence of zVAD-fmk, a caspase inhibitor (R&D systems, Minneapolis, MN, USA) (50 µM). Cells (1×10^4/well) were seeded in 96-well plates and incubated for 24 hours. To ensure the inhibitory effect, cells were pre-incubated with the inhibitor for 1 hour, and then, mAb 8B6 was added for 24 hours. After 24 hours of incubation, the number of viable cells was analyzed in the MTT assay as described in the Materials and Methods section.

Western blot analysis

Cells (5×10^5 cells) were seeded into 6-well culture plates and treated with mAb 8B6 (50 µg/ml) for 24 hours. Treated cells were washed twice with ice-cold phosphate-buffered saline, and then lysed using appropriate amount of lysis buffer (20 mM Tris–HCl, pH 7.4, 1% Nonidet P-40, 0, 25% DOC, 0, 15 M NaCl, 0, 1% SDS, 1 mM EDTA, phosphatase and protease inhibitors). Equal amounts of protein (20 µg) were separated on SDS–polyacrylamide gel, and electrotransferred to the polyvinylidene difluoride membrane (PVDF, Millipore, Billerica, MA, USA). Membranes were incubated with 5% milk-phosphate-buffered saline for 1 hour, washed with phosphate-buffered saline containing 0.05% Tween 20, and then incubated with monoclonal antibody reactive with cleaved-caspase-3 (clone 5A1E, Cell Signaling Technology Inc., Beverly, MA), with caspase-3 (clone 8G10, Cell Signaling Technology Inc.), and ß-actin (Clone C4, Millipore, Darmstadt, Germany). Secondary horseradish peroxidase-conjugated goat anti-mouse and monoclonal antibody and goat anti-rabbit (Jackson) were used for detection of bound primary antibody and bands were visualized by enhanced chemiluminescence (GE Healthcare, Velizy-Villacoublay, France). The chemiluminescence was analyzed by Fusion FX system (Vilber Lourmat, Eberhardzell, Germany) and Fusion software.
